# TWEAK: A New Player in Obesity and Diabetes

**DOI:** 10.3389/fimmu.2013.00488

**Published:** 2013-12-30

**Authors:** Joan Vendrell, Matilde R. Chacón

**Affiliations:** ^1^Research Unit, Centro de Investigación Biomédica en Red de Diabetes y Enfermedades Metabólicas Asociadas (CIBERDEM), Hospital Universitari de Tarragona Joan XXIII, Institut d’Investigació Sanitària Pere Virgili, Universitat Rovira i Virgili, Tarragona, Spain

**Keywords:** TWEAK, obesity, type 2 diabetes, adipose tissue, TNFα, insulin resistance, inflammation

## Abstract

Obesity and type 2 diabetes (T2D) are associated with chronic low-grade inflammation. Mounting evidence suggests the involvement of an inflammatory switch in adipose tissue, both in mature adipocytes and immune-competent cells from the stromal vascular compartment, in the progression of obesity and insulin resistance. Several inflammatory cytokines secreted by obese adipose tissue, including TNFα and IL-6 have been described as hallmark molecules involved in this process, impairing insulin signaling in insulin-responsive organs. An increasing number of new molecules affecting the local and systemic inflammatory imbalance in obesity and T2D have been identified. In this complex condition, some molecules may exhibit opposing actions, depending on the cell type and on systemic or local influences. Tumor necrosis factor weak inducer of apoptosis (TWEAK), a cytokine of the tumor necrosis (TNF) superfamily, is gaining attention as an important player in chronic inflammatory diseases. TWEAK can exist as a full-length membrane-associated (mTWEAK) form and as a soluble (sTWEAK) form and, by acting through its cognate receptor Fn14, can control many cellular activities including proliferation, migration, differentiation, apoptosis, angiogenesis, and inflammation. Notably, sTWEAK has been proposed as a biomarker of cardiovascular diseases. Here, we will review the recent findings relating to TWEAK and its receptor within the context of obesity and the associated disorder T2D.

## Introduction

A characteristic feature of obesity is a low-grade level of inflammation, likely originating in the expanding adipose tissue, which is illustrated by infiltration of immune cells, including macrophages, lymphocytes, and leukocytes (1). Pro-inflammatory cytokines released by activated immune cells and adipocytes can impair insulin signaling in insulin-responsive organs, promoting systemic insulin resistance, which increases the risk of developing type 2 diabetes (T2D) (2).

Tumor necrosis factor alpha (TNFα) was the first described cytokine to have a relevant role in obesity and an associated insulin-resistant state. Studies first conducted by Hotamisligil et al. (3), demonstrated an upregulation of TNFα in adipose tissue from obese patients. Since then, many members of the TNF superfamily have been shown to participate in obesity related diseases, including TNF-related apoptosis inducing ligand (TRAIL) (4), B cell activating factor (BAFF) (5), Lymphotoxin-α (LTα) (6, 7), Lymphotoxin β receptor (LTβR) (8), and Tumor necrosis factor ligand superfamily member 14 (TNFSF14) (9) among others. Recently, Tumor Necrosis Factor Weak Inducer of Apoptosis (TWEAK) has gained attention as a potentially important regulator of the inflammatory/anti-inflammatory equilibrium which takes place in the insulin-resistant milieu. TWEAK is a cytokine belonging to the TNF superfamily and triggers multiple, and often seemingly conflicting, cellular activities in a wide variety of cells, ranging from proliferation to cell death (10, 11). Like most TNF members, TWEAK protein exists as a membrane-bound (mTWEAK) form, and also as a soluble variant (sTWEAK), formed after proteolytic cleavage by a furin endoproteinase. Both forms are biologically active and can bind to Fibroblast growth factor-inducible 14 (Fn14), its only *bona fide* signal transducing receptor (12). Studies *in vitro* suggests that mTWEAK, can function as a juxtacrine signaling molecule and sTWEAK can elicit qualitatively different states of activity through Fn14 (12–14). Moreover, some authors have proposed sTWEAK as a potential biomarker in human cardiovascular diseases (CVDs) (15).

The cytoplasmic domain of Fn14 contains a TNF Receptor-Associated Factor (TRAF) binding site allowing recruitment of TRAF adapter proteins. This interaction is shared by most members of the TNF receptor family including TNFα, and plays a pivotal role in activating the nuclear factor κB (NF-κB) or mitogen-activated protein kinase (MAPK) pathway, which can be also activated by TWEAK (16). In particular, TRAF2 is implicated in the activation of TWEAK signaling in several human cell lines (17).

Interestingly, new data from human studies points to the TWEAK/Fn14 axis as a component of the network that contributes to the inflammatory imbalance occurring in insulin resistance-associated diseases (18–22). Here we review the role of TWEAK in adipocyte biology, and the prognostic and diagnostic value of its soluble form sTWEAK, within the context of insulin-resistant associated diseases, obesity, and diabetes.

## TWEAK and Adipocyte Biology

Tumor necrosis factor weak inducer of apoptosis mRNA expression was initially described in multiple human tissues (23). However, the first data concerning the expression of this cytokine in adipose tissue revealed mRNA gene expression of both *TWEAK* and its receptor *Fn14* in human fat depots from subcutaneous and visceral origin (18, 19, 24). Furthermore, altered patterns of TWEAK and Fn14 protein expression were observed in subcutaneous adipose samples from severely obese subjects when compared to healthy subjects (21).

In addition to adipocytes, adipose tissue contains a heterogeneous population of cells including preadipocytes, mesenchymal stem cells (MSC), endothelial cells, and macrophages among others cell types (25). Together, this collection of cells is termed the stromal vascular fraction (SVF). From this compartment, TWEAK expression has been detected mainly on the surface of macrophages and lymphoid cells (21, 26), whereas Fn14 expression has been detected in mature adipocytes (18, 21, 27, 28), preadipocytes (28, 29), MSC (29), and endothelial cells (30).

It is well recognized that Fn14 is a highly inducible receptor (11). Inflammation can regulate the expression of Fn14 in adipocytes and TWEAK in macrophages (19). In agreement with this data, isolated adipocytes from severely obese subjects exposed to a striking inflammatory environment, displayed increased levels of Fn14 (18, 21). Independently of inflammation, hypoxic stress is suggested to be a contributing factor in the adipocyte metabolism in the setting of obesity (31). Insufficient oxygen supply can lead to endoplasmic reticulum (ER) stress and mitochondrial dysfunction, and hypoxia alters the balance between pro- and anti-inflammatory activities in adipose tissue (32, 33). Although an up-regulation of the TWEAK/Fn14 axis, in parallel with hypoxia and ER-specific genes has been observed in the adipose tissue of severely obese subjects, studies emulating hypoxia and ER stress *in vitro* do not report any change in *TWEAK/Fn14* gene expression in isolated adipocytes or macrophages (19).

The pro-inflammatory milieu facilitated by hypoxia in adipose tissue may represent an important stimulus for macrophage attraction (34, 35). These cells may have a different polarized state (termed M1 for the pro-inflammatory and M2 for the anti-inflammatory subtypes) (35). Although there is a controversy regarding the balance between M1 and M2 macrophages in human obesity, and in obese mice models (36), many authors agree that the M2 type constitute the most abundant infiltrating macrophages found in human obesity (37, 38). In SVF from obese subjects mTWEAK protein has been found over-expressed. Since macrophages are an important component of the SVF cells, this finding points to macrophages as one of the cells that expresses this cytokine in the adipose tissue of obese subjects. In this regard, *in vitro* studies describe a higher level of mTWEAK expression in M2 human monocyte derived-macrophages when compared to M1 cells (21).

Tumor necrosis factor weak inducer of apoptosis stimulus may induce a pro-inflammatory activity in several human cell types including endothelial, kidney, synoviocytes, and muscle cells, among others (11). In human adipocytes, TWEAK stimulus alone resulted in a modest pro-inflammatory state, with up-regulation of the cytokines IL-6 and MCP-1, whereas leptin and adiponectin expression were unaltered (19, 28). This inflammatory effect seemed to be mediated through both canonical and non-canonical NF-κB pathway activation. Whereas the canonical pathway was activated in subcutaneous adipocytes, the non-canonical pathway appeared activated only in visceral adipocytes (21, 22). Furthermore, a moderate induction of the MAPKs ERK1/2 and p38 after TWEAK stimulus, has also been observed in both subcutaneous and visceral adipocytes (19, 22).

It is known that TWEAK can interfere with the differentiation ability of several cell types, including myogenic, osteoblast, chondrocyte, and erythroblast lineages (11). In addition, TWEAK can also inhibit adipocyte differentiation at an early stage, as indicated by a rapid reduction of the key adipogenic transcription factors Peroxisome proliferator-activated receptor gamma (PPARγ) and CCAAT/Enhancer Binding Protein α (C/EBPα) (28, 39).

In contrast to its effects on the differentiation capacity of adipocytes, TWEAK does not influence the metabolic function of these cells, such as glucose uptake and lipolysis. Furthermore, distinct from its clear apoptotic effect in neurons, monocytes, and tumor cell lines (40), a TWEAK stimulus does not induce apoptosis in adipocytes (22, 28).

Overall, the main impact of TWEAK on adipocytes appears to be an inhibition of adipocyte differentiation and the induction of a moderate inflammatory response.

## Circulating Levels of sTWEAK in Obesity and Diabetes

Diabesity is a new term coined to describe the common clinical associations between obesity and T2D, and highlights the close relationship between both states and their shared pathophysiological mechanisms. Chronic and subtle inflammation is usually documented in both states and can markedly influence cardiovascular (CV) outcomes (41).

Since the observation of lower levels of sTWEAK in patients with atherosclerosis and a negatively correlation between circulating sTWEAK levels and the intima/media thickness in asymptomatic subjects (15), decreased sTWEAK levels have been confirmed in many other CVD indications (40, 42–51). Consistent with these findings, obese individuals also present a similar trend of decreased sTWEAK in peripheral blood (21). Reinforcing the hypothesis that low sTWEAK levels associate with a poor CV profile, circulating levels of sTWEAK have also been found negatively associated with levels of glucose, glycated hemoglobin (HbA1), and also insulin resistance index (HOAM-IR) and central obesity; all of which are well known CV risk factors (20, 21, 46, 52). In contrast, the atherogenic lipid profile does not show a clear inverse association with circulating sTWEAK, and opposing data are reported in the literature. Whereas some studies describe a negative relationship with total cholesterol and triglycerides in severe obesity (21), other authors describe either a positive relationship with triglycerides (53) or no association at all (45). In severe obesity, changes in the levels of Free Fatty Acids were found to negatively influence circulating sTWEAK, indicating that lipotoxicity could be a modulator of sTWEAK levels. The observation of a lower rate of release of sTWEAK in carotid atheroma plaques, compared to normal arteries, supports a link between the lipotoxic effects of abnormal lipid accumulation and TWEAK synthesis (15).

Some of the evidence mentioned may lead to speculation about the potential anti-inflammatory behavior of sTWEAK, at least in high-risk atherogenic conditions. Indeed, the inverse relationship with inflammatory markers or surrogate inflammatory scores lends support to this hypothesis (20, 53). The rise of circulating sTWEAK levels after massive weight loss in severely obese subjects reinforces the parallelism between expression of this cytokine and other well recognized anti-inflammatory molecules such as adiponectin (21). Along similar lines, a trend toward positive correlation between levels of sTWEAK and adiponectin in patients on chronic hemodialysis has been reported (52).

Recently, a new study highlighted the relevance of decreased serum sTWEAK as a predictive marker of T2D. Interestingly this study was conducted in a high CV risk population, in which the incidence of T2D was assessed during a follow up (54). In this large prospective nested case-control study lower sTWEAK serum levels were found in incident cases compared to matched controls. Indeed, previous cross-sectional studies have also proposed a link between sTWEAK concentration and T2D (52).

The rationale that low levels of sTWEAK, in contrast to other cytokines, appears as protective in conditions with high CV risk diseases associated with an increased of chronic inflammatory activity, is incompletely understood. Several conceivable explanations have been proposed. A reduction of sTWEAK in serum, due to uptake by the Fn14 receptor has been postulated. Endothelial dysfunction is the initial pathophysiological step in the progression of vascular damage that precedes and leads to clinically visible CVD (55). Under these conditions, Fn14 expression is increased in the endothelium. Recently, we reported increased Fn14 expression in human adipocytes from severely obese subjects. These cells also showed an increase in Fn14 expression after inflammatory stimulation, thus increasing the availability for sTWEAK ligand, which could lead to a peripheral reduction of serum sTWEAK (19, 21).

An alternative hypothesis proposes the involvement of CD163, a monocyte-macrophage surface receptor which has been suggested to act as a scavenger receptor for sTWEAK (56). Soluble CD163 (sCD163) is a macrophage-specific serum marker which is elevated in inflammatory conditions (57). Circulating levels of sCD163 and sTWEAK are expressed in an opposite trend in human carotid atherosclerotic plaques (58). Moreover, CD163-expressing macrophages can bind and internalize sTWEAK *in vitro* (58). Thus, the reduction of sTWEAK could be related to the presence of sCD163, which is up-regulated both in patients with chronic kidney disease (CKD), and in obese subjects (51, 59–61). This incremental increase could enable sTWEAK degradation by inflammatory macrophages, leading to decreased sTWEAK levels, represented by the reduction in the sTWEAK/sCD163 ratio observed in some diseases such CKD (51). Thus, low sTWEAK levels may be related to the degree of macrophage activation. However, these observations are not fully coincident in TD2 patients. High serum levels of sCD163 have been reported as a useful predictive biomarker of T2D (62), but a more recent study reveals no association between circulating sCD163 and the incidence of T2D (54).

In contrast to the hypothesis of the potential anti-inflammatory behavior of sTWEAK, animal studies with different approaches to investigate the role of TWEAK/Fn14 axis in the development and progression of atherosclerosis, gain of function, or loss of function, have showed that TWEAK participates in the atherogenic process (63, 64) indicating that the “net” effect of the pathway is damaging rather than protective in this condition.

## sTWEAK Modulates TNFα Activity

To date, TNFα has focused the attention as a preponderant inflammatory cytokine with important implications both at local and systemic levels in obesity and related diseases. The action of TNFα on adipose tissue can alter the production of many adipokines, and this is relevant for the systemic effects of TNFα on insulin sensitivity and whole body energy homeostasis (65). sTWEAK and TNFα co-exist within the obese adipose tissue milieu, and both cytokines have a pro-inflammatory potential, although at the same concentrations TNFα is a much more potent and rapid inflammatory mediator than TWEAK (66).

Examination of the biological mechanisms through which sTWEAK improves insulin sensitivity has demonstrated that, in visceral adipocytes, treatment with sTWEAK ameliorates TNFα-induced insulin resistance on glucose uptake. This occurs by abolishing the stimulatory effect of TNFα on JNK1/2 kinase, which is directly involved in the development of insulin resistance (67). This effect is produced, at least in part, through a reduction in the cellular concentration of TRAF2, leading to a curbing of TNFα intracellular signaling events. Furthermore, this modulation of TNFα-induced changes in insulin sensitivity was found to be associated with an increase in the activity of PP2A, a Ser/Thr protein phosphatase known to negatively regulate cytokine signaling (22). Additionally, in human subcutaneous adipocytes, sTWEAK exerts a modulator effect over TNFα-induced cytokine production by inhibiting the MAPK and NF-κB signaling cascades commonly used by TNFα (21).

This protective/modulatory effect of sTWEAK on TNFα activity has been observed in different cell types such cultured fibroblast like synoviocytes obtained from synovial tissues of rheumatoid arthritis patients (68), in mouse cerebral cortical neurons (69), and also in several tumor human epithelial cell lines (70), suggesting a broader and general competitive behavior between sTWEAK and TNFα.

## Concluding Remarks

The duality between inflammatory and anti-inflammatory activity seems to be one of the major elements in the evolution of high CV risk diseases, such as obesity and T2D. In this scenario, some molecules may display contradictory actions, depending on the cell type and the location, and on the systemic, or local influence.

**Figure 1:**
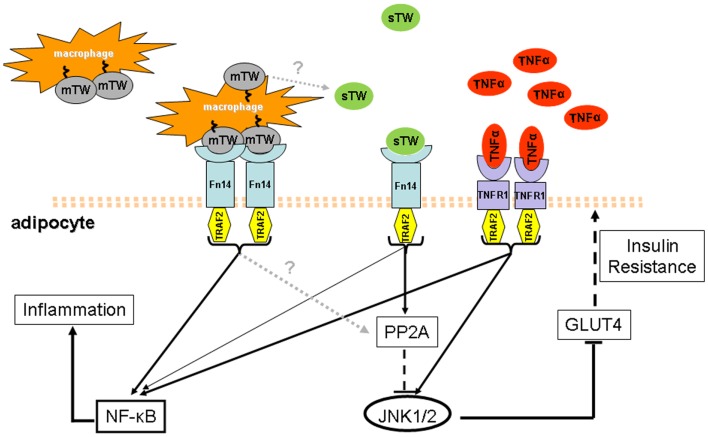
**Model of the state of the art of  TWEAK and  TNFα in obesity and  T2D conditions**. In obesity and T2D states, higher circulating levels of  TNFα and lower levels of sTWEAK have been observed. Additionally, obese adipose tissue is highly infiltrated with macrophages that could be a source of sTWEAK production and also express mTWEAK on the surface. The latter may establish a strong pro-inflammatory response in adipocytes where the Fn14 receptor is over-expressed and activates the NF-κB pathway. In contrast, sTWEAK has been identified as a negative regulator of  TNFα signaling since both signal via similar  TNFR-associated factors, including  TRAF2. Amelioration of  TNFα-induced insulin resistance by sTWEAK in obesity and  T2D could be a consequence, at least in part, of its direct regulation of JNK1/2 phosphorylation, controlled by PP2A phosphatase which is known to negatively regulate cytokine signaling. PPA2 activation could thereby be linked to the protective role of sTWEAK during the development of insulin resistance. Thus it is tempting to speculate that in obesity and  T2D, the increase of mTWEAK and the decrease of sTWEAK may help to maintain the pro-inflammatory effect of  TNFα-driven response.

Here we have summarized emerging data on the role of TWEAK within the context of metabolic inflammation. Despite the moderate inflammatory activity of the sTWEAK cytokine in adipocytes, a competitive interference ability of sTWEAK on TNFα signaling in the adipocyte has been revealed. Contrary to that observed with TNFα in obese and T2D patients, circulating sTWEAK appears as a protective element under these conditions. Interestingly, mTWEAK and sTWEAK have been shown to have different effects on signal transduction pathways. Since mTWEAK appears to be mainly expressed in macrophages, the metabolic effects of TWEAK may therefore differ in cells having contact with macrophages (e.g., adipocytes in an obesity context) and in more distant cells living in a macrophage-free environment. Thus, it is tempting to speculate that the decrease in sTWEAK levels, together with an increase of mTWEAK, may help to maintain the local pro-inflammatory effect of the TNF-α-driven response in obese and T2D conditions (Figure 1). Hence, the use of recombinant sTWEAK or Fn14 agonists to manipulate the TWEAK/Fn14 axis offers an exciting perspective for the treatment of insulin resistance and should be explored further.

## Conflict of Interest Statement

The authors declare that the research was conducted in the absence of any commercial or financial relationships that could be construed as a potential conflict of interest.

## Abbreviations

BMI, body mass index; CD163, cluster of differentiation 163; CVDs, cardiovascular disease; ERK, extracellular signal-regulated kinases; Fn14, fibroblast growth factor-inducible 14; HOMA-IR, homeostasis model assessment of insulin resistance; IL-6, interleukin 6; MAPKs, mitogen-activated protein kinases; mTWEAK, full-length TWEAK isoform; NF-κB, nuclear factor kappa-light-chain-enhancer of activated B cells; p38, mitogen-activated protein kinase; PP2A, protein phosphatase 2A; sTWEAK, soluble TWEAK isoform; SVF, stromal vascular fraction; T2D, type 2 diabetes; TNFα, tumor necrosis factor alpha; TRAF2, TNF receptor-associated factor 2; TWEAK, tumor necrosis factor weak inducer of apoptosis.
